# Using static method to measure tolmetin solubility at different pressures and temperatures in supercritical carbon dioxide

**DOI:** 10.1038/s41598-020-76330-9

**Published:** 2020-11-11

**Authors:** Mahboubeh Pishnamazi, Samyar Zabihi, Pegah Sarafzadeh, Fatemeh Borousan, Azam Marjani, Rasool Pelalak, Saeed Shirazian

**Affiliations:** 1 Institute of Research and Development, Duy Tan University, Da Nang, 550000 Viet Nam; 2 The Faculty of Pharmacy, Duy Tan University, Da Nang, 550000 Viet Nam; 3 Department of Chemical Sciences, Bernal Institute, University of Limerick, Limerick, Ireland; 4 Department of Process Engineering, Research and Development Department, Shazand-Arak Oil Refinery Company, Arāk, Iran; 5 Department of Chemical Engineering, Faculty of Al’Zahra Shiraz, Fars Branch, Technical and Vocational University, Shiraz, Iran; 6 Department of Chemistry, Yasouj University, Yasouj, 75914-353 Iran; 7 Incubation Centre of Arak Science and Technology Park, Fanavari Atiyeh Pouyandegan Exir Company, Arāk, 381314-3553 Iran; 8 Incubation Centre of Arak Science and Technology Park, Fanavari Arena Exir Sabz Company, Arāk, 381314-3553 Iran; 9 Department for Management of Science and Technology Development, Ton Duc Thang University, Ho Chi Minh City, Viet Nam; 10 Faculty of Applied Sciences, Ton Duc Thang University, Ho Chi Minh City, Viet Nam; 11 Chemical Engineering Faculty, Sahand University of Technology, P.O. Box 51335-1996, Sahand New Town, Tabriz, Iran; 12 Laboratory of Computational Modeling of Drugs, South Ural State University, 76 Lenin prospekt, Chelyabinsk, Russia 454080

**Keywords:** Medical research, Molecular medicine, Chemistry

## Abstract

Tolmetin is a non-steroidal anti-inflammatory drug being used to decrease the level of hormones which are the reasons for pain, swelling, tiredness, and stiffness for osteoarthritis and rheumatoid arthritis cases. We evaluated its solubility in supercritical carbon dioxide (SC-CO_2_) with the aim of drug nanonization, considering temperature and pressure variations between 120 and 400 bar and 308–338 K, in the experiments. In this way, a PVT solubility cell based on static solubility approach coupled with a simple gravimetric procedure was utilized to evaluate the solubility of tolmetin. The solubility values between 5.00 × 10^−5^ and 2.59 × 10^−3^ mol fraction were obtained for tolmetin depending on the pressure and temperature of the cell. The measured data demonstrated a direct correlation between pressure and solubility of tolmetin, while the effect of temperature was a dual effect depending on the crossover pressure (160 bar). The calculated solubility data were modeled using several semi-empirical correlations, and the fitting parameters were calculated using the experimental data via appropriate optimization method. The correlated solubility data revealed that the KJ model was the most accurate one with an average absolute relative deviation percent (AARD%) of 6.9. Moreover, the carried out self-consistency analysis utilizing these correlations illustrated great potential of these models to extrapolate the solubility of tolmetin beyond the measured conditions.

## Introduction

In general, it is claimed that using non-steroidal anti-inflammatory drugs (NSAIDs) can enhance the risk of heart attack or a stroke for the people who take these drugs unfortunately without warning. Basically, the NSAIDs rise the probability risks of significant cardiovascular thrombotic problems, myocardial infarction, and stroke with a direct correlation to the duration and dosage of its use. Tolmetin, taken for osteoarthritis, rheumatoid arthritis, ankylosing spondylitis and juvenile rheumatoid arthritis, is one among the approved NSAIDs. Tolmetin is a drug from the acetic-acid derivative class similar to sulindac, diclofenac, and indomethacin^1,2^. Unfortunately, similar to the other drugs of NSAIDs family, tolmetin is totally well-tolerated, with some side effects such as headache, and dizziness. Moreover, some significant problems can originate from NSAIDs usage such as gastrointestinal ulceration and bleeding, increased risk for cardiovascular disease, renal dysfunction, and hypersensitivity reactions including anaphylaxis, exfoliative dermatitis, and Stevens-Johnson syndrome^2^. Respecting these facts, it seems necessary to enhance the efficacy of this drug concomitant with lowering its required dosage.

One of the efficient ways to justify these purposes is the micronization/nanonization process in order to prepare drug particles with small particle size and high surface area^3^. In detail, if a drug introduces a low solubility in water, it can be subjected to a micronization process to reduce its size to a value below 10 µm^3^ since according to Noyes–Whitney model, particle size reduction can substantially enhance the drug bioavailability of poorly soluble substances if their size reduce to micron or nano level^4,5^. Moreover, as the drug particles reduce to values below 5 µm, a thinner diffusion layer around the particles would be established and the rate of drug absorption is not affected anymore by the hydrodynamics in the gastrointestinal tract leading to more effectiveness of the drug in the body^6,7^. Respecting these facts, several micronization processes are proposed during the past decades among which, using supercritical fluid (SCF)-based technologies mostly utilize carbon dioxide (CO_2_) as the main solvent, is one of the most recently proposed methods to micronize different drugs including diclofenac^8^, sulindac^9^, fenoprofen^10^, piroxicam^11^, etc.

Regardless of the utilized methods, all of them require the drug solubility in supercritical solvents at various operational conditions since these parameters can indicate the required size of the equipment or other operating conditions. In this way, several works have been done for measuring solubility of potential drugs in supercritical carbon dioxide such as phenylepherin hydrochloride^12^, sulindac^13^, fluoxetine hydrochloride^14^, trioctylmethyl ammonium^15^, piroxicam^16^, etc.^17–21^. Unfortunately, since measuring the experimental values of the solubility for all of the substances at the wide range of temperature and pressure is impossible, it is highly required to utilize models and correlations which can simulate solubility values of them under various pressures and temperatures such as equations of state (EOS)^22,23^, artificial neural network^24,25^, and, semi-empirical density based correlations^26,27^. Among the possible modeling approaches, semi-empirical density based correlations are one of the widely utilized and examined model since, (a) the required mathematical approach for solving these correlations is mostly simple which only needs multiple regression methods, and (b) they just need pressure, temperature, and density values at the corresponding pressure and temperature which are quickly and accurately measurable. So, there is no need for estimated parameters during the modeling approach to make them more reliable and accurate compared with the aforementioned methods. These correlations have been used and validated in literature for prediction of solubility^28,29^.

Considering these facts, the solubility of tolmetin in SC-CO_2_ using a PVT cell linked with well-known gravimetric technique is measured under wide ranges of pressures (120–400 bar) and temperatures (308–338 K) for the first time since there is no report regarding the solubility of this drug in the literature. This drug was selected as the sample drug since there is no report regarding its solubility in SC-CO_2_ according to the best knowledge of the authors. Besides, the calculated solubility results were correlated using five different three-parameter semi-empirical correlations which are density-based thermodynamic models mainly used for solubility predictions. These models include: Bartle et al.^30^, Garlapati and Madras^27^, Mendez-Santiago and Teja (MST)^31^, Chrastil^32^, and Kumar-Johnston (KJ)^33^. For this purpose, a multiple regression method was used to find the fitting parameters of each correlation making anyone to reproduce and correlate the solubility of tolmetin in various pressures and temperatures. Finally, the extrapolative capability of these correlations was examined using self-consistency test since this capability is an undeniable and favorable potential of any predictive method.

## Experimental

### Materials

Tolmetin (C_15_H_15_NO_3_), CAS number of 26171-23-3, with molecular weight of 257.29 g gmol^−1^ was selected as the sample drug for solubility measurement in the current investigation. Tolmetin was purchased from *Matrix Scientific* (> 95% purity) and was also further treated using CO_2_ (purity > 99.8%) at 338 K and 500 bar to purify it^34^.

### Solubility tests

**Figure 1:**
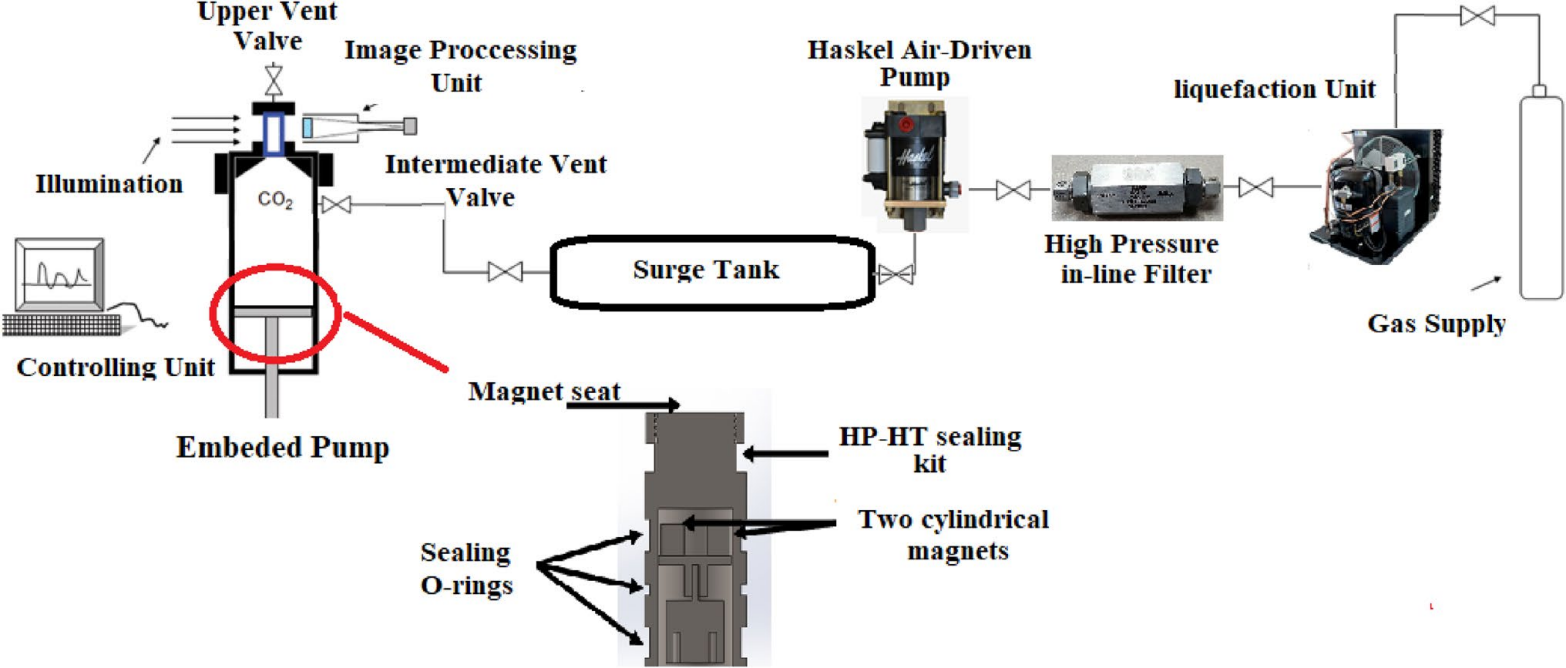
SC-CO_2_ drug solubility apparatus^19^. Reprinted from^19^, Copyright (2020), with permission from Elsevier.

A solubility test apparatus was designed and employed in this work to measure the solubility values of tolmetin at different pressures and temperatures. The apparatus was designed by *Fanavari Atiyeh Pouyandegan Exir Company* (Arak, Iran), and is shown in Fig. 1. The applicability and reliability of the system has been validated in our previous publication for measuring Fenoprofen solubility in supercritical CO_2_^34^. The system operates based on gravimetric method in which the weight of sample before and after the test is measured to obtain the values of solubility. As shown in Fig. 1, the heart of process is the PVT cell (volume = 0.4 L) which is used for the solubility tests at various temperatures and pressures using the designed control system connected to the cell. In the liquefaction unit of the process, supercritical CO_2_ is generated and sent to PVT cell after passing through the booster, filtering, and surge tank units. All experiments were conducted in triplicate, and the average values are reported. The detailed description of procedure and solubility measurements can be found elsewhere^18,19,21,34^.

## Results and discussion

### Experimental results

In measuring the solubility of tolmetin, eight different pressures (120 to 400 bar, interval 40 bar) were set, while the cell temperatures were set at four values, i.e. 308, 318, 328, and 338 K. The measurements were repeated three times, and the statistical analysis indicated that the highest value of relative standard deviation was 8.6%, and the average relative standard deviation of 6.2% was calculated, confirming the acceptable level of reproducibility of the measurements.

**Figure 2:**
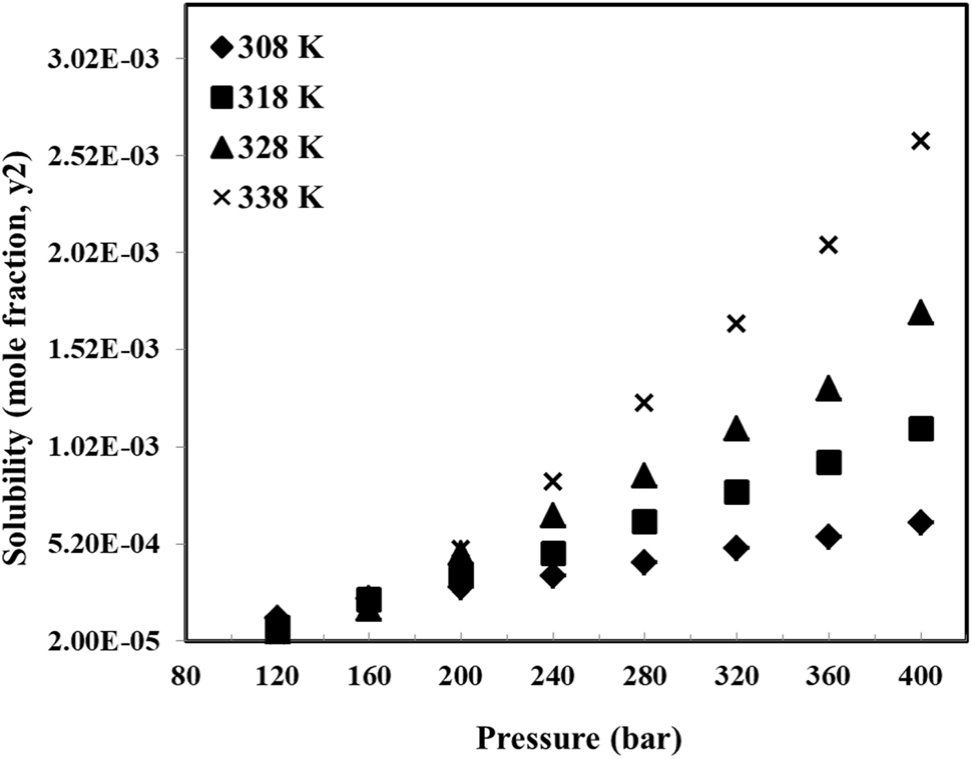
Solubility values of tolmetin.

The experimental solubility data of tolmetin is shown in Fig. 2 and listed in Table 1 along with standard deviation (SD) values for the measurements. It turned out that the tolmetin solubility values lie between 5.00 × 10^−5^ and 2.59 × 10^−3^ mol fraction (see Table 1). It is clearly observed that the tolmetin solubility is a strong function of temperature and pressure. The effect of pressure on solubility is shown to be increasing, however sharper change is observed at higher temperatures, e.g. 338 K. On the other hand, the effect of temperature is initially decreasing, and then increasing after a certain point (around 160 bar). This point is known as cross-over point in which the influence of temperature on tolmetin solubility changes. In fact, as PVT cell pressure is kept below 160 bar, an enhancement in the cell temperature caused a reduction in tolmetin solubility while in the pressures greater than this value, enhancing temperature may more effectively activate the sublimation pressure modification which can compensate the density decrement effect due to temperature enhancement, consequently causes more solubility of tolmetin. Furthermore, the observation of cross-over pressure point in the solubility data confirm the reliability of data for tolmetin solubility, as reported by Foster et al.^35^.

**Table 1:** Solubility of tolmetin.

P/bar	T/K							
	308		318		328		338	
	y	SD	y	SD	y	SD	y	SD
120	1.39 × 10^−4^	1.20 × 10^−5^	8.83 × 10^−5^	5.06 × 10^−6^	6.80 × 10^−5^	1.46 × 10^−6^	5.00 × 10^−5^	2.68 × 10^−6^
160	2.39 × 10^−4^	1.89 × 10^−5^	2.35 × 10^−4^	1.05 × 10^−5^	1.86 × 10^−5^	5.32 × 10^−6^	1.82 × 10^−4^	4.99 × 10^−6^
200	2.97 × 10^−4^	1.51 × 10^−5^	3.65 × 10^−4^	2.21 × 10^−5^	4.69 × 10^−4^	3.28 × 10^−5^	4.93 × 10^−4^	2.32 × 10^−5^
240	3.55 × 10^−4^	1.89 × 10^−5^	4.70 × 10^−4^	2.56 × 10^−5^	6.64 × 10^−4^	4.16 × 10^−5^	8.41 × 10^−4^	5.69 × 10^−5^
280	4.21 × 10^−4^	2.84 × 10^−5^	6.34 × 10^−4^	4.73 × 10^−5^	8.74 × 10^−4^	6.98 × 10^−5^	1.24 × 10^−3^	7.65 × 10^−5^
320	4.98 × 10^−4^	2.34 × 10^−5^	7.85 × 10^−4^	5.52 × 10^−5^	1.18 × 10^−3^	1.44 × 10^−5^	1.65 × 10^−3^	1.11 × 10^−4^
360	5.53 × 10^−4^	4.44 × 10^−5^	9.40 × 10^−4^	6.44 × 10^−5^	1.32 × 10^−3^	7.81 × 10^−5^	2.06 × 10^−3^	1.72 × 10^−4^
400	6.30 × 10^−4^	5.40 × 10^−5^	1.11 × 10^−3^	7.17 × 10^−5^	1.71 × 10^−3^	1.09 × 10^−4^	2.59 × 10^−3^	2.06 × 10^−4^

Standard uncertainty (u), are u (T) = 0.1 K and u (P) = 0.35 bar.

### Modeling

**Figure 3:**
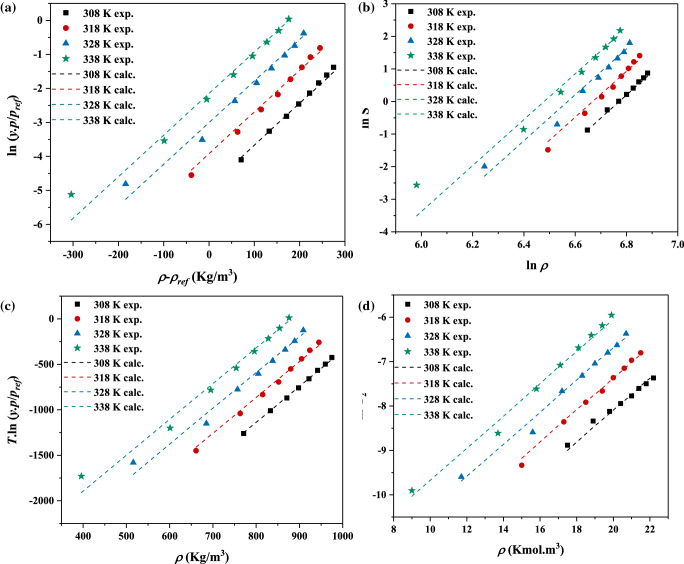
Tolmetin solubility results applying, (**a**) Bartle et al. model, (**b**) Chrastil, (**c**) MST and (**d**) KJ model.

The finding results of solubility are correlated via five semi-empirical thermodynamic equations for predicting tolmetin solubility. The modelling results are shown in the Table 2 and Fig. 3. Measuring tolmetin solubility values applying the fitting parameters listed in Table 2 for all models, demonstrated the modeling possibility of the solubility results of tolmetin include AARD% of about 11.3%, 10.2%, 6.9%, 12.3% and 12.1% for Bartle et al., Mendez-Santiago-Teja, Kumar and Johnstone (KJ), Chrastil, and Garlapati and Madras models, respectively.

**Table 2:** Fitting parameters of semi-empirical density-based correlations.

Models (AARD%)^a^	Mathematical equation of model	Constants		
		a	b	c
Bartle et al. (11.3%)	$$\ln \left( {\frac{y \cdot p}{{p^{ref} }}} \right) = a + {\raise0.7ex\hbox{$b$} \!\mathord{\left/ {\vphantom {b {T/K}}}\right.\kern-\nulldelimiterspace} \!\lower0.7ex\hbox{${T/K}$}} + c \cdot (\rho - \rho_{ref} )$$	25.9	− 9480	0.0122
Mendez-Santiago-Teja (10.2%)	$T \cdot \ln \left( {\frac{y \cdot p}{{p^{ref} }}} \right) = a + b \cdot T/K + c \cdot \rho /{\text{kg}} \cdot {\text{m}}^{ - 3}$	12,662-	27.205	3.93
Kumar and Johnstone (6.9%)	$$\ln y = a + {\raise0.7ex\hbox{$b$} \!\mathord{\left/ {\vphantom {b {T/K}}}\right.\kern-\nulldelimiterspace} \!\lower0.7ex\hbox{${T/K}$}} + c \cdot \rho \, /{\text{kmol}} \cdot {\text{m}}^{ - 3}$$	7.71	− 7094.4	0.361
Chrastil (12.3%)	$$\ln s/{\text{kg}} \cdot {\text{m}}^{ - 3} = a + {\raise0.7ex\hbox{$b$} \!\mathord{\left/ {\vphantom {b {T/K}}}\right.\kern-\nulldelimiterspace} \!\lower0.7ex\hbox{${T/K}$}} + c \cdot \ln \rho /{\text{kg}} \cdot {\text{m}}^{ - 3}$$	− 7149.3	24.404	7.03
Garlapati and Madras (12.1%)	$\ln {\text{y}} = {\text{a}} + {\text{b}}/{\text{T}}/{\text{K}} + {\text{c}}\ln (\uprho /{\text{kg}} \cdot {\text{m}}^{ - 3} \,{\text{T}}/{\text{K}})$	− 66.58	− 5202	6.025

^a^AARD% = 100 × ∑ ((y^calc^ − y^exp^)/y^exp^).

A considerable advantage of the examined models especially Bartle et al., and Chrastil model is their ability to predict the total enthalpy, enthalpy of vaporization and solvation enthalpy as follow:

1 $$ \Delta H_{sub} = - Rb \to 78.8\,{\text{kJ/mol}} $$

2 $$ \Delta H_{total} = - Ra \to 59.4\,{\text{kJ/mol}} $$

3 $$ \Delta H_{solvation} = \Delta H_{total} - \Delta H_{sub} \to - 19.4\,{\text{kJ/mol}} $$

*R* is the gas universal constant.

**Figure 4:**
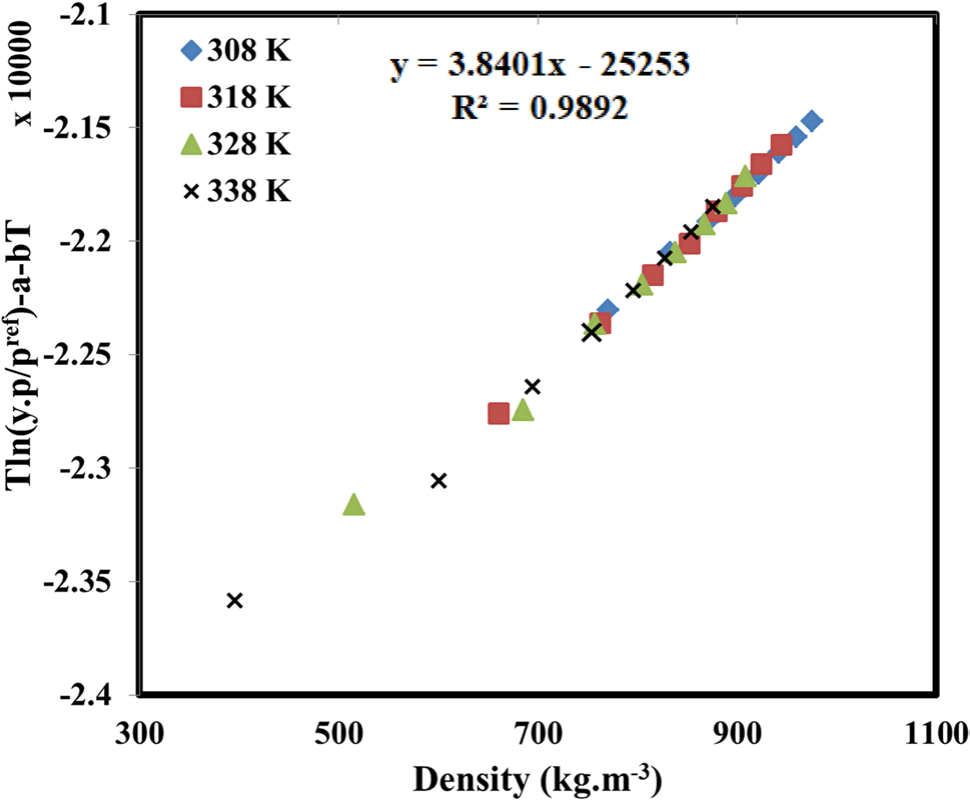
Self-consistency test utilizing MST model.

Besides the correlative capability, extrapolative potential is the other important advantage of any correlation or model. Respecting that, the extrapolative capability of the examined models was investigated performing self-consistency test (see Figs. 3 and 4). The results demonstrated that the solubility results could satisfy the linear behavior for all the examined isotherms and isobars in the current investigation regardless of their values. So, it can be concluded that it would be possible to estimate the solubility of tolmetin in the temperatures and pressures out of the calculated range since the solubility data obey a simple linear behavior.

## Conclusions

Measuring tolmetin solubility in SC-CO_2_ at various pressures and temperatures is the main objective of the current investigation. Then, a static approach linked to a simple gravimetric approach was utilized to calculate the solubility of tolmetin with the assist of PVT cell. The measurement revealed that tolmetin solubility was between 5.00 × 10^−5^ and 2.59 × 10^−3^ based on mole fraction. The measurements demonstrated that it is possible to enhance the solubility of tolmetin by increasing pressure, while increasing temperature can lead to two different trends of increasing and decreasing. In detail, since the temperature can have influence on density and sublimation pressure concomitantly, but act in opposite directions, it would be the net of these two challenging factors which demonstrated that temperature with decreasing or increasing result. The point where the temperature influences changes called cross-over pressure which for the current the system was a transient region between 120–160 bar where for the pressures of 120 and 160 bar, decreasing the density because of the increasing the temperature is dominant while for the pressures upper than 160 bar, sublimation pressure modification would be dominant leading to an enhancement in the solubility of tolmetin in SC-CO_2_. Besides the experimental measuring of tolmetin solubility, the measured values were correlated utilizing five density-based correlations since it would not be possible to calculate the solubility of tolmetin in all the ranges of pressure and temperature. The correlation results revealed that among the examined models, KJ led to the lowest AARD% value of 6.9% while the other models leading higher AARD%, although they still introduce a great admissible precision. The other important privilege is the extrapolative ability of these models which can be examined by performing self-consistency tests. The performed self-consistency test revealed a great extrapolative capability of these models since the measured solubility data for all the examined temperatures and pressures justify a straight line that shows the other thermodynamic conditions beyond the considered ranges can justify the current line which means the extrapolative ability of these models.
